# Biomechanical Effect of Disc Height on the Components of the Lumbar Column at the Same Axial Load: A Finite-Element Study

**DOI:** 10.1155/2022/7069448

**Published:** 2022-10-25

**Authors:** Jae-Gyeong Jeong, Sungwook Kang, Gu-Hee Jung, Mingoo Cho, Hyunsoo Kim, Kyoung-Tae Kim, Dong-Hee Kim, Jong-Moon Hwang

**Affiliations:** ^1^Department of Rehabilitation Medicine, Kyungpook National University Hospital, Daegu 41944, Republic of Korea; ^2^Precision Mechanical Process and Control R&D Group, Korea Institute of Industrial Technology, Jinju-si, Gyeongsangnam-do 52845, Republic of Korea; ^3^Department of Orthopaedic Surgery, Gyeongsang National University, College of Medicine, Gyeongsang National University Changwon Hospital, 11 Samjeongja-ro, Seongsan-gu, Changwon-si 51472, Republic of Korea; ^4^Department of Neurosurgery, Kyungpook National University Hospital, Daegu 41944, Republic of Korea; ^5^Department of Neurosurgery, School of Medicine, Kyungpook National University, Daegu 41944, Republic of Korea; ^6^Department of Orthopaedic Surgery, Gyeongsang National University, College of Medicine, Jinju-si, Gyeongsangnam-do 52727, Republic of Korea; ^7^Department of Rehabilitation Medicine, School of Medicine, Kyungpook National University, Daegu 41944, Republic of Korea

## Abstract

Intervertebral discs are fibrocartilage structures, which play a role in buffering the compression applied to the vertebral bodies evenly while permitting limited movements. According to several previous studies, degenerative changes in the intervertebral disc could be accelerated by factors, such as aging, the female sex, obesity, and smoking. As degenerative change progresses, the disc height could be reduced due to the dehydration of the nucleus pulposus. This study aimed to quantitatively analyze the pressure that each structure of the spine receives according to the change in the disc height and predict the physiological effect of disc height on the spine. We analyzed the biomechanical effect on spinal structures when the disc height was decreased using a finite-element method investigation of the lumbar spine. Using a 3D FE model, the degree and distribution of von-Mises stress according to the disc height change were measured by applying the load of four different motions to the lumbar spine. The height was changed by dividing the anterior and posterior parts of the disc, and analysis was performed in the following four motions: flexion, extension, lateral bending, and axial rotation. Except for a few circumstances, the stress applied to the structure generally increased as the disc height decreased. Such a phenomenon was more pronounced when the direction in which the force was concentrated coincided with the portion where the disc height decreased. This study demonstrated that the degree of stress applied to the spinal structure generally increases as the disc height decreases. The increase in stress was more prominent when the part where the disc height was decreased and the part where the moment was additionally applied coincided. Disc height reduction could accelerate degenerative changes in the spine. Therefore, eliminating the controllable risk factors that cause disc height reduction may be beneficial for spinal health.

## 1. Introduction

Intervertebral discs are fibrocartilage structures that play a role in buffering the compression applied to the vertebral bodies evenly while permitting limited movements. Intervertebral discs could be structurally divided into annulus fibrosus, the outer part of the disc, and nucleus pulposus, the inner part of the disc. Annulus fibrosus is mainly composed of multiple layers of collagen type 1 fibers that run obliquely between vertebral bodies, and the nucleus pulposus is composed of proteoglycan and water gel that are loosely connected by collagen type 2 and elastin fibers [1].

Although the cause or process of degenerative changes in the intervertebral disc is not fully understood, they can occur especially when the disc does not receive sufficient nutrients [2]. In addition, degenerative changes are promoted after the disc gets damaged by external stimuli, such as spinal surgery [3–5]. According to several previous studies, degenerative changes in the intervertebral disc could be accelerated by factors such as aging, female sex, obesity, and smoking [6–9]. As degenerative change progresses, the height of the disc also gets reduced due to the dehydration of the nucleus pulposus [2]. Furthermore, degenerative changes in the disc can cause annulus cleft and tear, endplate damage, and osteophyte formation [10–12].

A series of disc degenerative changes can cause the nucleus pulposus to bulge radially outward, which can mechanically compress and irritate the nerve root [13]. In addition, nociceptive nerves exist in the annulus fibrosus and facet joint regions, which can be irritated by tensile stress applied to the annulus fibrosus and facet joint narrowing [14, 15]. Therefore, lower back pain and radiating pain may occur due to the preceding causes, which may decrease the patient's quality of life [16].

Although there have been several previous studies regarding the symptoms that can occur due to a reduction in disc height and degenerative changes, quantitative analysis studies on the change in pressure on spinal structures, according to a decrease in disc height, are rare.

Measurement through *in vivo* studies is the most consistent with reality. However, because of the invasive method, not only can it cause unwanted harm to healthy individuals or patients, such as accelerating the degeneration process, but it can also cause difficultly in setting the desired disc height [17]. In addition, even when using a cadaver, the biophysical environment of a living person cannot be completely reproduced [18, 19].

These limitations can be overcome by using the finite-element modeling (FEM) [20–22]. The use of FEM can reduce harmful results to patients by avoiding an invasive process and can provide economic benefits due to low cost. Therefore, this study aimed to quantitatively analyze the pressure that each structure of the spine receives according to the change of the disc height and predict the physiological effect of disc height on the spine using FEM.

## 2. Materials and Methods

In this study, an analysis was performed using a three-dimensional (3D) FEM to study the effect of disc height change on the vertebral spine and disc. The stress distribution was observed by applying the load of four different motions to the lumbar spine.

### 2.1. Development of the FE Model

**Figure 1:**
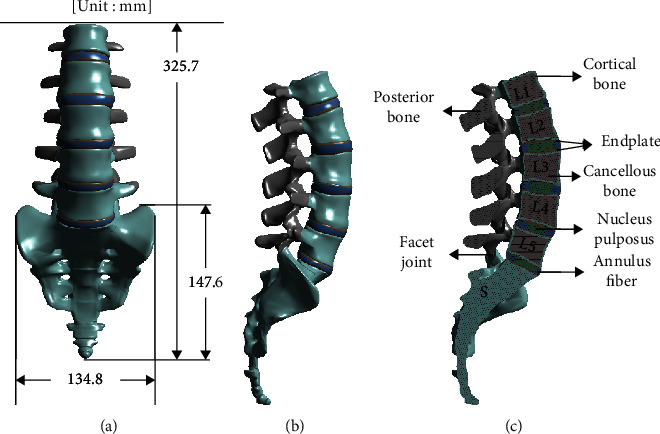
Finite-element (FE) model. (a) Front view of the lumbar spine model, (b) side view of the lumbar spine model, and (c) cross-section of the whole model.

In this study, the analysis of a 3D FEM for the vertebral spine was performed. The 3D FEM includes intervertebral discs (including nucleus pulposus and annulus fibrosus), endplates, and facet joints, as well as a lumber spine of L1 to L5 (including cortical bone, cancellous bone, and posterior element). In addition, a sacrum was modeled to apply structural boundary conditions. Computed tomography data were converted into 3D solid modeling using the ANSYS SpaceClaim software, resulting in a 3D model capable of final finite element analysis, as shown in Figure 1.

**Figure 2:**
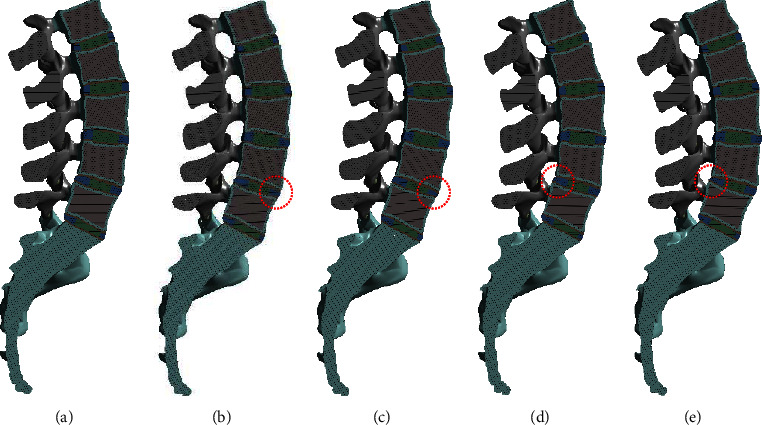
Modification of disc height. (a) Normal disc height, (b) 75% of the anterior disc height, (c) 50% of the anterior disc height, (d) 75% of the posterior disc height, and (e) 50% of the posterior disc height.

To identify the effect of disc height change, the height of the disc located between L4 and L5 was changed (Figure 2). The models with an anterior height of 75% and 50% and a posterior height of 75% and 50% were compared to a normal disc (100%).

### 2.2. Mesh Information and Material Properties for the FE Model

To perform a 3D finite element analysis for each motion, the structure was meshed using the Static Structural module of ANSYS Workbench. The mesh size of each structure was set to 2–5 mm, and an element type of second-order tetrahedron (10 nodes) was used. The mesh size was determined using the following method. First, we compared the von-Mises stress values derived from the mesh size of *N* mm and *N* − 1 mm. At this time, when the stress difference was less than 2%, the mesh size was determined as *N* mm. Then, the above-mentioned sensitivity analysis was performed for each structure in which the mesh size was decreased by 1 mm starting from 5 mm. In conclusion, the mesh size of each structure was finally determined. Since the disc height change was caused by a degenerative change regardless of bone density, the material properties of a healthy individual were applied to the FE model.  Table 1 shows data on the mesh and material properties of structures for finite element analysis [23–25].

### 2.3. Loading and Boundary Conditions

**Figure 3:**
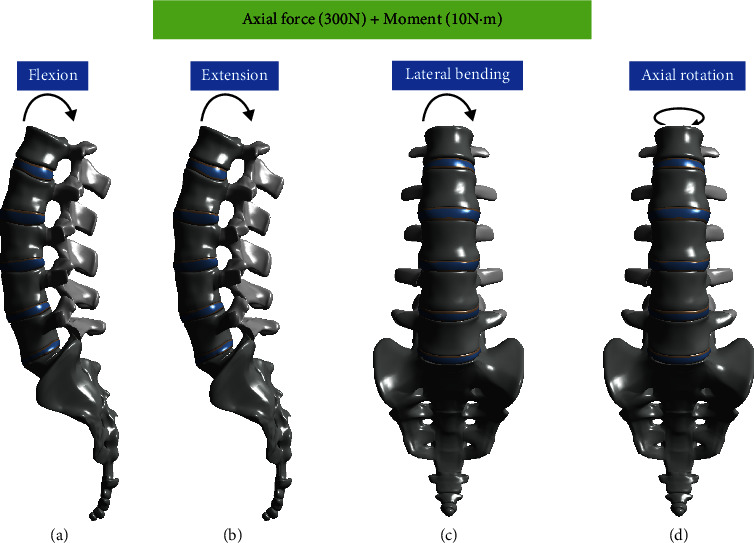
Four different motions implemented by simultaneously applying axial force and moment. The axial force applied a load of 300 N to the upper surface of L1. A moment of 10 N·m was applied to trigger the motions of (a) flexion, (b) extension, (c) lateral bending, and (d) axial rotation.

For the finite element analysis, four different motions were implemented by simultaneously applying axial force and moment. The axial force applied a load of 300 N to the upper surface of L1. A moment of 10 N m was applied to trigger the motion of flexion, extension, lateral bending, and axial rotation (Figure 3). All structures were connected to each other in bonding contact conditions, and all degrees of freedom for the sacrum were constrained.

## 3. Results

We observed how the load applied to each structure of the L45 level vertebrae (cortical bone, cancellous bone, posterior bone, endplate (upper and lower), and disc (outer and inner)) changed according to the moment while decreasing the height of the anterior and posterior parts of the disc. Tables 2–5 show the absolute values of the total load applied to the spinal structure for each situation and the rate of change of the load at the disc height, which is reduced by 50% compared to normal. A positive value denoted by an asterisk indicates that a larger load than normal represents that the disc height is reduced by 50%.

### 3.1. Von-Mises Stress of Spine FEM with Anterior Disc Modification

By reducing the height of the anterior disc to 75% and 50%, compared to normal, we examined how the stress applied to the L4-5 level spinal structure changed at moments of flexion, extension, lateral bending, and axial rotation. First, when the disc height was reduced to 50% in the flexion mode, the stress applied to all structures was increased, except for the L5 cancellous bone and L4 posterior bone (Table 2). Second, in the extension mode, the stress applied to all structures was reduced, except for the L4 and L5 cancellous bones (Table 3). Third, in the lateral bending mode, the stress applied to the L45 cortical bone and cancellous bone, L5 posterior bone, and L45 lower endplate was increased (Table 4). Finally, in the axial rotation mode, the stress applied to L5 cortical bone, L45 cancellous bone, L45 lower endplate, and L45 disc was increased (Table 5).

**Figure 4:**
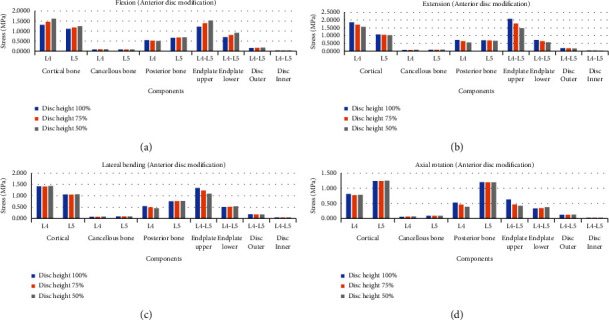
Von-Mises stress comparison according to changes in the anterior disc height at the lumbar spine in four different motions. (a) Flexion mode, (b) extension mode, (c) lateral bending mode, and (d) axial rotation mode.

When the graph was analyzed (Figure 4), as the height of the anterior disc decreased, the load tended to increase in the flexion mode, and the load tended to decrease in the extension mode. In the case of lateral bending and axial rotation, there was no clear trend according to the change in the height of the anterior disc, but an increase in stress was observed in 6 of 10 structures.

### 3.2. Von-Mises Stress of Spine FEM with Posterior Disc Modification

**Figure 5:**
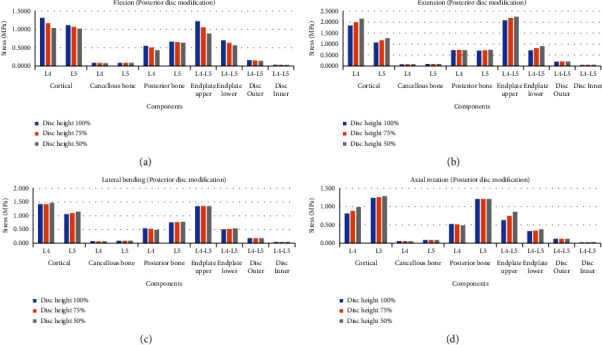
Von-Mises stress comparison according to change of the posterior disc height at the lumbar spine in four different motions. (a) Flexion mode, (b) extension mode, (C) lateral bending mode, and (d) axial rotation mode.

By reducing the height of the posterior disc to 75% and 50% compared to normal, we examined how the stress applied to the L45 level spinal structure changed at moments of flexion, extension, lateral bending, and axial rotation. First, when the disc height was reduced to 50% in the flexion mode, the stress applied to all structures except for the L5 cancellous bone was reduced (Table 2). Second, in the extension mode, the stress applied to all structures increased (Table 3). Third, in the lateral bending mode, the stress applied to all structures, except for the L4 cancellous bone and L4 posterior bone, was increased (Table 4). Finally, in the axial rotation mode, the stress applied to all structures, except for the L4 cortical bone, cancellous bone, and posterior bone, was increased (Table 5). When analyzing the graph (Figure 5), it was seen that as the height of the posterior disc decreased, the load in the flexion mode decreased, and the load in the extension mode increased. In the case of lateral bending and axial rotation, the load was generally increased compared to the load when the height of the anterior disc was decreased.

### 3.3. Stress Distribution in Each Spinal Component

**Figure 6:**
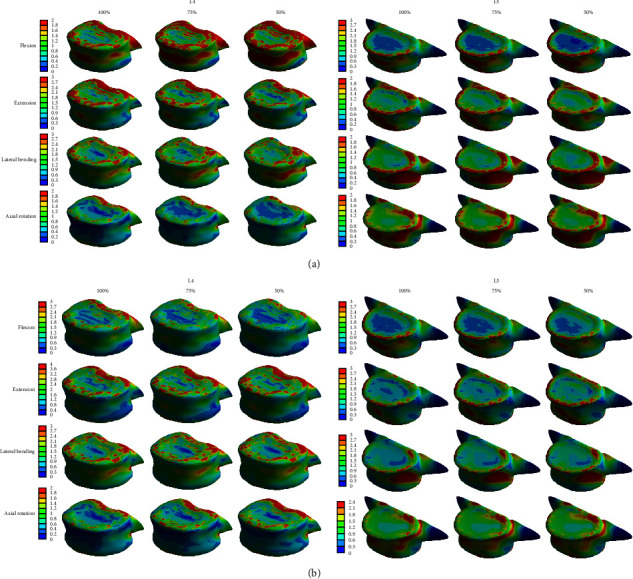
Von-Mises stress distribution on the cortical bone of L4–5 in four different motions. (a) Change in anterior disc height and (b) change in posterior disc height; unit: MPa.

**Figure 7:**
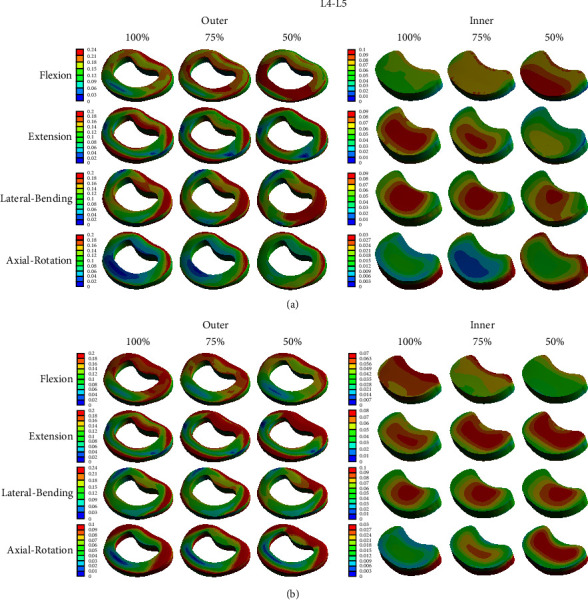
Von-Mises stress distribution on the intervertebral disc of L4–5 in four different motions. (a) Change in anterior disc height and (b) change in posterior disc height; unit: MPa.

**Figure 8:**
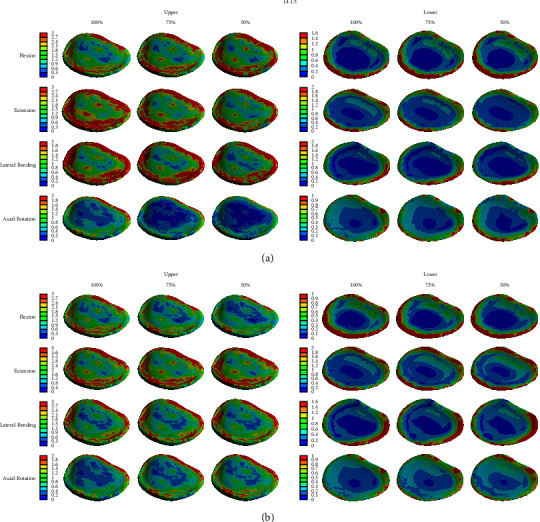
von-Mises stress distribution on the intervertebral endplate of L4–5 in four different motions. (a) Change in anterior disc height and (b) change in posterior disc height; unit: MPa.

Although the sum of the stresses applied to each structure of the spine is important, it is possible to perform a more accurate analysis by evaluating the stress distribution by considering the portion where the stress increases locally according to the height of the disc. Figures 6–8 show the stress distribution of the cortical bone, intervertebral disc, and endplate. When the disc height and moment were changed, the distribution of stress appeared similar at each moment. The load increased toward the edge of the cortical bone, outer disc (annulus fibrosus), and endplate and was concentrated in the center of the inner disc (nucleus fibrosus). However, as the height of the disc decreased, the pattern of change in the magnitude of the stress slightly differed. As the height of the anterior disc and the posterior disc decreased, the local stress increased (increased in the red area). However, as the anterior disc height decreased, the local stress decreased in the extension and lateral bending modes. Conversely, as the posterior disc height decreased, the local stress decreased in the flexion mode.

## 4. Discussion

Unlike in previous studies, the current study changed the disc height in the anterior and posterior parts and analyzed the change in stress accordingly. A large part of the nucleus pulposus of the disc is composed of proteoglycan, and chondroitin sulfate of proteoglycan contains water. Due to these structural features, the nucleus pulposus of the disc exhibits viscoelastic behavior [26, 27]. From the perspective of the overall lifespan, the nucleus pulposus ages slowly over a long period, leading to a decrease in proteoglycan. This leads to dehydration and a permanent change in the material properties of tissue [28, 29]. However, from the perspective of the purpose of this study, the viscoelastic behavior of the disc is close to that of an elastic body that restores to its original shape when the external load is removed. In other words, this study was not interested in the behavior considering the viscoelasticity of the intervertebral disc but only focused on the stress applied to the adjacent vertebral body as the height of the disc decreased.

Based on the data analyzed using FEM, we found that as the height of the disc decreased, the stress applied to the vertebral structures under various vertebral movements with the same load increased. When the anterior disc height was decreased, the stress increased in the flexion mode, whereas when the posterior disc height was decreased, the stress increased in the extension mode. Conversely, a marked stress reduction was observed when the anterior disc height was decreased in the extension mode and when the posterior disc height was decreased in the flexion mode. As shown from the previous results, the increase in stress was more prominent when the part where the disc height was decreased and the part where the moment was additionally applied coincided. On the other hand, there was no clear trend when the anterior disc height decreased in the lateral bending and axial rotation modes, and the stress tended to increase when the posterior disc height decreased.

Since the results in Tables 2–5 are obtained by calculating the sum of stresses applied to each structure, there is a limitation in that, that is, it cannot reflect the increase in the load in a specific part, as shown in Figures 6–8. Therefore, it may be helpful to analyze both the numerical values and stress distributions. According to the results, a decrease in disc height can increase the stress applied to the spinal structure, but a decrease in the posterior disc height generates a slightly larger amount of stress in the spinal structure than in the anterior disc height; therefore, the adverse effect on the spinal physiology is also greater. In the process of degenerative changes in the spine, it is rare that only one side of the disc is reduced in height. However, pathological changes, such as disc bulging and herniation, usually occur in the posterolateral direction. The reasons for this are as follows: the posterior longitudinal ligament is narrower and weaker than the anterior longitudinal ligament [30] and the thickness of the annulus fibrosus in the posterior direction is thinner [31]. Therefore, it is easy to decrease the posterior disc height compared to decreasing the anterior disc height. In this situation, the degenerative change can be accelerated by the increase in the stress applied to the spinal structure.

Similar to the results of the current study, several studies also showed that the von-Mises stress or shear stress applied to the annulus fibrosus tends to increase as disc degeneration progresses [32–34]. However, existing studies were different in terms of the application of the comprehensive concept of disc degeneration, reflecting disc height and structural changes, specifically in structural properties and osteophyte formation. In addition, unlike in previous studies, the current study analyzed the stress applied to various structures composing the spine and observed how the value changes depending on the disc height. Therefore, considering the differences from previous studies, the current study presented valuable information.

In our analysis of the distribution of von-Mises stress applied to the disc using FEM, except for bony structures, stress was highest in the endplate, which connects the vertebral body and the intervertebral disc. Additionally, the magnitude of the stress increased toward the edge of each structure. This result seemed to be due to the expansion of the nucleus pulposus to the outside by compressive force, as the nucleus pulposus has a large expansion rate, and tensile stress is additionally applied to the edge of the structure. Lu et al. [16] also reported the tendency of the stress to increase in the posterolateral direction of the disc. However, the changing pattern of the stress distribution according to the disc height was not clearly observed. The area with higher stress is similar to the edge of the vertebral body, where osteophyte frequently occurs, and near the endplate, where degenerative changes of the spine begin.

As the disc height decreases, the stress applied to the spinal structure changes, and the disc height and the facet joint space become proportional, as reported by a previous study [35]. According to this result, the facet joint space becomes narrower as the disc height decreases, which may increase the possibility of causing facet joint osteoarthritis and facet joint pain. Another study reported that the degenerative changes in the disc reduce the density of the trabecular core in vertebral bone [36]. This means that the decrease in disc height can also act as a factor to increase the occurrence of compression fracture. Therefore, the decrease in disc height is expected to accelerate degenerative changes in the spine by increasing the stress applied to the spinal structures and can negatively affect the spinal structures in various ways.

Because of developments in the medical field, the elderly population has increased, and as a result, diseases that can frequently occur in the elderly are becoming an issue. A representative example is chronic low back pain (cLBP) caused by degenerative changes in the spine, which has the highest prevalence worldwide among chronic pain conditions [37]. It is important to note that cLBP causes not only pain but can also become an economic concern, reducing the patient's quality of life. Degenerative changes in the spine are a concept that encompasses disc degeneration, facet joint osteoarthritis, vertebral body degeneration, and ligament degeneration [38]. Among them, the nucleus pulposus of the intervertebral disc is the structure in which the onset of degenerative changes most frequently occurs [2].

Although the specific mechanism that causes degenerative changes in the disc remains unelucidated, one hypothesis is the lack of nutrition to the disc [39]. The end plate is mainly involved in supplying nutrients to the disc [40]. If damage is applied to the end plate due to causes such as excessive load or trauma, the nutrient supply may be insufficient, and degenerative changes of the disc may be induced [41, 42].

One of the phenomena caused by degenerative changes in the disc is a decrease in the disc height. With the aging process, the proteoglycan and water content of the nucleus pulposus decreases, and consequently, the height of the disc decreases [8]. Factors associated with a decrease in disc height include not only aging but also smoking, surgery, sex, and being overweight [6–9]. According to previous studies, nicotine, a major component in products consumed by smokers, can promote degenerative changes in the disc by reducing blood flow around the intervertebral disc through vasoconstriction [43, 44]. Spinal surgery, such as discectomy, induces a series of cellular, structural, and functional changes in the intervertebral disc, which reduces intradiscal pressure and consequently decreases disc height [3]. Previous studies have reported that sex hormones influence disc degeneration. Estrogen acts on the proliferation of disc cells and promotes disc degeneration in estrogen deficiency conditions, such as menopause [45–47]. Moreover, an increase in body weight affects the disc height by increasing the axial load applied to the spinal structures [7]. Symptoms such as osteophyte formation, endplate sclerosis, and cleft and fissure of the disc may appear as degenerative changes that progress with the decrease in disc height [42]. These structural changes in spinal components could cause back pain and further neurological symptoms by directly compressing the nerve root through disc bulging or herniation or by stimulating the nociceptive nerve in the annulus of the disc [1].

To delay the acceleration of degenerative changes in the spine caused by this cycle, it is necessary to prevent the decrease in disc height in advance. Among the aforementioned factors that affect disc height reduction, it is important to improve correctable factors. Interventions, such as smoking cessation, weight loss, and avoiding postures that overload the spine, may be helpful. According to results from a previous study, an increase in the paraspinal muscle volume could reduce the load applied to the spinal structure [48]. Therefore, strengthening of the paraspinal muscle through appropriate exercise also seems to be helpful in preventing disc degeneration. In patients who underwent spinal surgery, degenerative changes are likely to be accelerated compared with those in the healthy individual. Therefore, it is necessary to enable patients to form a desirable lifestyle through appropriate education on healthy spines from an early stage.

There are several limitations in this study. First, in FEM, each spinal structure was analyzed through fixed material properties. In the intervertebral disc, as degenerative changes progressed, not only the height of the disc decreased but material properties also changed as the water content of the nucleus pulposus decreased and became closer to a solid-like material [10]. However, the information was insufficient, and for the efficiency of the analysis, changes in material properties were neglected from the analysis. Second, the 3D-FE model did not fully reflect all the structures of the spine, such as ligaments and muscles. Since some structures were not included to increase the efficiency of the analysis, there may be differences from the actual spine biomechanics. Third, only the lower lumbosacral vertebra (L45) was included in this study, and the upper lumbar spine (L1–3) was not included. However, since most spinal degenerative changes are concentrated at the lower spine level (L4–5, S1) [49], our model is also thought to have significance. In the future, if studies that improve the abovementioned limitations are performed, the biomechanical action of the actual spine can be predicted more accurately in degenerative conditions.

## 5. Conclusions

Intervertebral discs are fibrocartilage structures, which play a role in buffering the compression applied to the vertebral bodies evenly while permitting limited movements. According to several previous studies, degenerative changes in the intervertebral disc could be accelerated by factors, such as aging, the female sex, obesity, and smoking. As degenerative change progresses, the disc height could be reduced due to the dehydration of the nucleus pulposus. In this study, we analyzed the changes in the stress applied to the spinal structures as the disc height decreased. The analysis of a three-dimensional finite element model for the vertebral spine was performed. To identify the effect of disc height change, the disc height located between L4 and L5 was changed. The models with anterior height and posterior height of 75% and 50% were compared to a normal disc (100%). For finite element analysis, four different motions were implemented by simultaneously applying axial force and moment. The axial force applied a load of 300 N to the upper surface of L1. A moment of 10 N m was applied to trigger the motion of flexion, extension, lateral bending, and axial rotation. As the disc height decreased, the stress applied to the spinal structure generally increased. In particular, the results were clearer when the area where the disc height decreased and the area where the axial force was concentrated coincided. Also, the distribution of stress tended to increase toward the edge, except for the nucleus pulposus. The results of this study indicate that the decrease in disc height can also act as a factor that promotes degenerative changes in the spine. In conclusion, eliminating the controllable risk factors that cause disc height reduction may be beneficial for spinal health.

## Figures and Tables

**Table 1:** Information on mesh and material properties for the finite element model of the vertebral body.

Item (mesh size)			Number of nodes	Number of elements	Elastic modulus, E (MPa)	Poisson's ratio (*ν*)	Reference
Cortical bone (3 mm)			69,667	37,138	12,000	0.3	[23]
Cancellous bone (5 mm)			71,381	47,169	200	0.25	[23]
Posterior bone (4 mm)			37,963	20,990	3,500	0.25	[23]
Endplate (2 mm)			30,204	13,537	1,000	0.3	[23]
Facet joint (2 mm)			1,842	474	11	0.4	[24]

Nucleus pulposus (3 mm)	100%		346,396	239,611	1	0.49	[23]
	Posterior	75%	343,307	237,389			
		50%	339,608	234,645			
	Anterior	75%	342,039	236,368			
		50%	337,141	232,928			

Annulus fibrosus (3 mm)	100%		358,484	239,295	4.2	0.45	[23]
	Posterior	75%	351,189	233,959			
		50%	344,576	229,112			
	Anterior	75%	350,436	233,370			
		50%	341,288	226,898			

MPa, megapascal.

**Table 2:** The von-Mises stress on the structures of the L4–5 spine in the flexion mode when the disc height was reduced to 75% and 50% compared to normal (100%).

Component		The 50% anterior disc model, MPa (a)	The 75% anterior disc model, MPa (b)	The 100% anterior disc model, MPa (c)	Rate of change [(a-c)/c ∗ 100] (%)	The 50% posterior disc model, MPa (d)	The 75% posterior disc model, MPa (e)	The 100% posterior disc model, MPa (f)	Rate of change [(d-f)/f ∗ 100] (%)
Cortical bone	L4	1.6156	1.4583	1.3132	23.03^∗^	1.0364	1.1683	1.3132	−21.08
	L5	1.2441	1.1806	1.1147	11.61^∗^	1.0202	1.0689	1.1147	−8.48
Cancellous bone	L4	0.0983	0.0935	0.0870	13.01^∗^	0.0785	0.0835	0.0870	−9.82
	L5	0.0846	0.0878	0.0886	−4.48	0.0888	0.0888	0.0886	0.32^∗^
Posterior bone	L4	0.5045	0.5271	0.5514	−8.50	0.4352	0.5064	0.5514	−21.07
	L5	0.6889	0.6768	0.6636	3.80^∗^	0.6376	0.6526	0.6636	−3.91
Endplate (upper)	L4–5	1.5277	1.3928	1.2242	24.79^∗^	0.8875	1.0533	1.2242	−27.50
Endplate (lower)	L4–5	0.9118	0.8047	0.7028	29.75^∗^	0.5642	0.6352	0.7028	−19.72
Disc (outer)	L4–5	0.1805	0.1714	0.1598	12.97^∗^	0.1434	0.1517	0.1598	−10.25
Disc (inner)	L4–5	0.0388	0.0370	0.0338	14.79^∗^	0.0283	0.0309	0.0338	−16.09

^∗^ indicates that a larger load than normal was received when the disc height was reduced by 50%.

**Table 3:** The von-Mises stress on the structures of the L4‐5 spine in the extension mode when the disc height was reduced to 75% and 50% compared to normal (100%).

Component		The 50% anterior disc model, MPa (a)	The 75% anterior disc model, MPa (b)	The 100% anterior disc model, MPa (c)	Rate of change [(a-c)/c ∗ 100] (%)	The 50% posterior disc model, MPa (d)	The 75% posterior disc model, MPa (e)	The 100% posterior disc model, MPa (f)	Rate of change [(d-f)/f ∗ 100] (%)
Cortical bone	L4	1.5655	1.6992	1.8453	−15.16	2.1541	1.9900	1.8453	16.73^∗^
	L5	1.0263	1.0463	1.0692	−4.01	1.2675	1.1655	1.0692	18.55^∗^
Cancellous bone	L4	0.0816	0.0803	0.0809	0.92^∗^	0.0881	0.0853	0.0809	8.92^∗^
	L5	0.1007	0.0963	0.0926	8.66^∗^	0.0982	0.0953	0.0926	6.05^∗^
Posterior bone	L4	0.5631	0.6448	0.7204	−21.83	0.7227	0.7293	0.7204	0.32^∗^
	L5	0.6623	0.6795	0.6970	−4.97	0.7354	0.7169	0.6970	5.51^∗^
Endplate (upper)	L4-5	1.4776	1.7772	2.0771	−28.86	2.2418	2.1836	2.0771	7.93^∗^
Endplate (lower)	L4-5	0.5666	0.6428	0.7180	−21.08	0.8999	0.8127	0.7180	25.34^∗^
Disc (outer)	L4-5	0.1739	0.1857	0.1972	−11.79	0.2124	0.2095	0.1972	7.71^∗^
Disc (inner)	L4-5	0.0371	0.0438	0.0481	−22.80	0.0522	0.0516	0.0481	8.61^∗^

^∗^ indicates that a larger load than normal was received when the disc height was reduced by 50%.

**Table 4:** The von-Mises stress on the structures of the L4–5 spine in the lateral bending mode when the disc height was reduced to 75% and 50% compared to normal (100%).

Component		The 50% anterior disc model, MPa (a)	The 75% anterior disc model, MPa (b)	The 100% anterior disc model, MPa (c)	Rate of change [(a-c)/c ∗ 100] (%)	The 50% posterior disc model, MPa (d)	The 75% posterior disc model, MPa (e)	The 100% posterior disc model, MPa (f)	Rate of change [(d-f)/f ∗ 100] (%)
Cortical bone	L4	1.432	1.409	1.416	1.14^∗^	1.467	1.422	1.416	3.62^∗^
	L5	1.062	1.052	1.055	0.69^∗^	1.145	1.095	1.055	8.56^∗^
Cancellous bone	L4	0.083	0.078	0.074	12.24^∗^	0.071	0.073	0.074	−3.65
	L5	0.093	0.091	0.089	4.37^∗^	0.091	0.090	0.089	2.54^∗^
Posterior bone	L4	0.449	0.494	0.542	−17.29	0.485	0.522	0.542	−10.51
	L5	0.774	0.766	0.761	1.70^∗^	0.775	0.768	0.761	1.87^∗^
Endplate (upper)	L4–5	1.096	1.235	1.345	−18.51	1.346	1.354	1.345	0.04^∗^
Endplate (lower)	L4–5	0.540	0.513	0.507	6.36^∗^	0.544	0.519	0.507	7.19^∗^
Disc (outer)	L4–5	0.175	0.178	0.183	−3.94	0.187	0.186	0.183	2.53^∗^
Disc (inner)	L4–5	0.041	0.044	0.046	−10.23	0.046	0.047	0.046	0.34^∗^

^∗^ indicates that a larger load than normal was received when the disc height was reduced by 50%.

**Table 5:** The von-Mises stress on the structures of the L4–5 spine in the axial rotation mode when the disc height was reduced to 75% and 50% compared to normal (100%).

Component		The 50% anterior disc model, MPa (a)	The 75% anterior disc model, MPa (b)	The 100% anterior disc model, MPa (c)	Rate of change [(a-c)/c ∗ 100] (%)	The 50% posterior disc model, MPa (d)	The 75% posterior disc model, MPa (e)	The 100% posterior disc model, MPa (f)	Rate of change [(d-f)/f ∗ 100] (%)
Cortical bone	L4	0.781	0.772	0.809	−3.48	0.982	0.877	0.809	21.45
	L5	1.249	1.240	1.239	0.86^∗^	1.282	1.254	1.239	3.50^∗^
Cancellous bone	L4	0.069	0.064	0.059	17.71^∗^	0.053	0.056	0.059	−9.58
	L5	0.091	0.090	0.088	2.33^∗^	0.089	0.089	0.088	0.08^∗^
Posterior bone	L4	0.388	0.457	0.524	−25.98	0.488	0.513	0.524	−6.89
	L5	1.195	1.198	1.203	−0.74	1.205	1.205	1.203	0.12^∗^
Endplate (upper)	L4–5	0.420	0.466	0.629	−33.18	0.856	0.747	0.629	36.12^∗^
Endplate (lower)	L4–5	0.376	0.342	0.331	13.67^∗^	0.378	0.343	0.331	14.23^∗^
Disc(outer)	L4–5	0.125	0.122	0.118	5.88^∗^	0.122	0.121	0.118	3.26^∗^
Disc(inner)	L4–5	0.024	0.023	0.024	0.38^∗^	0.026	0.025	0.024	11.09^∗^

^∗^ indicates that a larger load than normal was received when the disc height was reduced by 50%.

## Data Availability

The data used to support the findings of this study are available from the corresponding author upon request.
